# Reliability, Factor Structure and Predictive Validity of the Widespread Pain Index and Symptom Severity Scales of the 2010 American College of Rheumatology Criteria of Fibromyalgia

**DOI:** 10.3390/jcm9082460

**Published:** 2020-07-31

**Authors:** Carmen M. Galvez-Sánchez, Pablo de la Coba, Stefan Duschek, Gustavo A. Reyes del Paso

**Affiliations:** 1 Department of Psychology, University of Jaén, 23071 Jaén, Spain; pcoba@ujaen.es (P.d.l.C.); greyes@ujaen.es (G.A.R.d.P.); 2 Institute of Psychology, UMIT—University for Health Sciences, Medical Informatics and Technology, 6060 Hall in Tirol, Austria; stefan.duschek@umit.at

**Keywords:** fibromyalgia, ACR criteria, chronic pain, depression, central pain sensitization

## Abstract

Fibromyalgia syndrome (FMS) is a chronic condition of widespread pain. In 2010, the American College of Rheumatology (ACR) proposed new diagnostic criteria for FMS based on two scales: the Widespread Pain Index (WPI) and Symptoms Severity (SS) scale. This study evaluated the reliability, factor structure and predictive validity of WPI and SS. In total, 102 women with FMS and 68 women with rheumatoid arthritis (RA) completed the WPI, SS, McGill Pain Questionnaire, Trait Anxiety Inventory, Fatigue Severity Scale, Oviedo Quality of Sleep Questionnaire, and Beck Depression Inventory. Pain threshold and tolerance and a measure of central sensitization to pain were obtained by pressure algometry. Values on WPI and SS showed negative-skewed frequency distributions in FMS patients, with most of the observations concentrated at the upper end of the scale. Factor analysis did not reveal single-factor models for either scale; instead, the WPI was composed of nine pain-localization factors and the SS of four factors. The Cronbach’s α (i.e., Internal consistency) was 0.34 for the WPI,0.83 for the SS and 0.82 for the combination of WPI and SS. Scores on both scales correlated positively with measures of clinical pain, fatigue, insomnia, depression, and anxiety but were unrelated to pain threshold and tolerance or central pain sensitization. The 2010 ACR criteria showed 100% sensitivity and 81% specificity in the discrimination between FMS and RA patients, where discrimination was better for WPI than SS. In conclusion, despite their limited reliability, both scales allow for highly accurate identification and differentiation of FMS patients. The inclusion of more painful areas in the WPI and of additional symptoms in the SS may reduce ceiling effects and improve the discrimination between patients differing in disease severity. In addition, the use of higher cut-off values on both scales may increase the diagnostic specificity in Spanish samples.

## 1. Introduction

Fibromyalgia syndrome (FMS) is a chronic disorder characterized by widespread and persistent musculoskeletal pain, accompanied by symptoms including fatigue, insomnia, depression, anxiety, and cognitive impairments [1]. While the etiology of FMS remains unknown, it is widely acknowledged that central pain sensitization and impairments in endogenous pain inhibitory mechanisms play a crucial role in its pathogenesis [2,3]. This is expressed in hyperalgesia and allodynia, which together characterize FMS; moreover, patients exhibit reduced thresholds and tolerance to evoked pain, increased responses during protocols measuring pain sensitization, and exaggerated activity in the neuromatrix of pain during painful stimulation [4,5,6,7,8,9].

An important problem in FMS research and its clinical management is the lack of objective markers of the disease and of reliable clinical measures for its diagnosis [10,11]. The subjective nature of FMS symptoms, along with the lack of objective indicators, hinders disease comprehension, effective healthcare, and medical and social acceptance. Based on previous research [12,13], in 1990 the American College of Rheumatology (ACR) established preliminary criteria for FMS [1]. However, these criteria have been repeatedly criticized due to the difficulty of using pressure algometry in primary healthcare, the low predictive validity of evoked pain with respect to clinical pain, and the lack of consideration of accompanying symptoms among other aspects [14,15]. 

In 2010, the ACR proposed new criteria, based on the Widespread Pain Index (WPI) and Symptom Severity (SS) scale [16]. The WPI includes a list of 19 painful body areas. Whereas the SS includes two parts: Part 2a calculates severity scores for fatigue, waking unrefreshed and cognitive symptoms. Part 2b derives a general symptom severity score from a list of 41 symptoms. For FMS diagnosis, one of two conditions must be fulfilled: WPI ≥ 7 and SS ≥ 5, or WPI between 3 and 6 and SS ≥ 9. As in the 1990 criteria, symptom duration of at least the three previous months was required. In 2011 and 2016, these diagnostic criteria were revised again [17,18]. 

The present study evaluated the reliability and predictive validity of the WPI and SS in a sample of FMS patients recruited with the 1990 ACR criteria. Its objectives were as follows: (1) estimation of the frequency distribution of WPI and SS scores in FMS patients and a sample of patients with rheumatoid arthritis (RA), as a chronic pain syndrome of peripheral origin. (2) Evaluation of the factor structure of WPI and SS. To the best of our knowledge, no previous studies have analyzed the underlying factorial structure of these scales. Based on their aims and composition [16], we hypothesized that both scales are unidimensional; accordingly, WPI items should load mainly on a painful body-part factor and SS items basically on a pain-severity factor. (3) Assessment of the internal consistency of the WPI and SS. Studies analyzing the internal consistency of both scales together reported Cronbach’s α values of 0.75 in Japanese [19] and 0.87 in French [20] samples. A Turkish study reported Cronbach´s α values of 0.78 for the WPI, 0.53 for SS and 0.77 for the two scales together [21]. (4) Evaluation of the predictive validity of the WPI and SS with respect to the core FMS symptoms; two studies demonstrated inverse associations of WPI and SS with measures of quality of life [22,23]. A previous Spanish study reported positive correlations of WPI and SS with fatigue and depression [23]. In the present study, relationships between WPI and SS scores with self-reported clinical pain, fatigue, insomnia, anxiety, depression, and pain catastrophizing were quantified. (5) Estimation of associations between WPI and SS scores, evoked pain measures (pain threshold and tolerance) and a marker of central pain sensitization. The most well-established hypothesis regarding FMS pathophysiology pertains to central nervous sensitization to pain [2,4]. However, to date, no study has analyzed the association of WPI and SS scores with parameters reflecting this phenomenon. (6) Evaluation of the diagnostic accuracy of the 2010 ACR criteria and WPI and SS scores to differentiate between FMS and RA patients. Objectives 2–5 were analyzed only in the FMS sample. 

## 2. Material and Methods

### 2.1. Participants

In total, 102 women with FMS, recruited from the Fibromyalgia Association of Jaén (Spain), participated in this study. All patients were examined by a rheumatologist and met the 1990 ACR criteria of FMS. The control group was comprised of 68 patients with RA who met the American Rheumatology Association (ARA) criteria for RA [24], with no previous diagnosis of FMS neither FMS features. All participants were right-handed. Exclusion criteria included the presence of metabolic abnormalities, neurological disorders, drug abuse, and severe somatic (e.g., cancer) or psychiatric (e.g., psychotic) diseases. 

### 2.2. Assessment Instruments

The patients´ clinical history and demographic data were obtained during a semi-structured interview. The Structured Clinical Interview for Axis I Disorders of the Diagnostic and Statistical Manual for Mental Disorders [25] was used to diagnose possible mental disorders. In addition, the following self-report questionnaires were administered:

The Spanish version [26] of 2010 ACR criteria of fibromyalgia [16]. The WPI and SS comprise a total of 64 items. The WPI includes a checklist of 19 possible painful areas (score range: 0–19). Patients are asked to indicate whether or not each point is painful. Part 2a of the SS includes 4-point Likert scales (0–3) to evaluate the severity of fatigue, cognitive symptoms, and waking unrefreshed; Part 2b consists of a checklist of 41 symptoms (dry eyes, fever, constipation, nausea, itching, etc.) Patients have to indicate whether they have these symptoms. Based on the total number of symptoms, patients are assigned one of the following scores: 0 symptoms, score of 0; 1 to 10 symptoms, score of 1; 11 to 24 symptoms, score of 2; and 25 or more symptoms, score of 3. The SS (score range: 0–12) is obtained by adding the scores for part 2a (score range: 0–9) and part 2b (score range: 0–3). 

The Spanish version of [27] McGill Pain Questionnaire (MPQ) [28]. This 73-item instrument allows quantification of the sensory, emotional and cognitive components of pain experience. In this study, the Sensory Pain (score range: 0–84), Emotional Pain (score range: 0–22), Current Pain Intensity (score range: 0–5), and Total Pain Experience (score range: 0–146) subscales were used. Cronbach´s α values between 0.56 (emotional pain) and 0.74 (total pain) were reported [27].

The Spanish version [29] of State-Trait Anxiety Inventory (STAI) [30]. This instrument enables the assessment of current and habitual anxiety levels (20 items per scale) by 4-point Likert scales (score range: 0–60). Values of Cronbach´s α are 0.93 for State Anxiety and 0.87 for Trait Anxiety [29].

The Spanish version [31] of Beck Depression Inventory (BDI) [32]. This 21-item scale was applied to assess the severity of symptoms of depression (4-point Likert scales, scores range: 0–63). The Cronbach´s α was reported as 0.95 [31].

The Spanish version [33] of Fatigue Severity Scale (FSS) [34]. This scale quantifies fatigue based on 9 items (7-point Likert scales, score range: 9–63). It has a Cronbach´s α of 0.88 [33].

Oviedo Quality of Sleep Questionnaire (COS) [35]. The Insomnia subscale of the instrument, comprising 9 items (5-point Likert scales, score range: 4–54) was used. The Cronbach´s α of this subscale was reported as 0.88 [35].

The Spanish version [36] of Coping Strategies Questionnaire (CSQ) [37]. The Catastrophizing scale (6-point Likert scales, score range: 0–36) was used, which has a Cronbach’s α of 0.89 [36].

### 2.3. Pain Induction and Quantification

Pressure algometry was used for pain evocation. While pain sensitivity was assessed by the traditional static measures of pain threshold and tolerance, central nervous pain sensitization was estimated using a dynamic pain protocol. For this purpose, sensitization to slowly repeated evoked pain (SREP) was used. SREP sensitization was reliably observed in FMS patients but not in healthy controls or RA patients [5,6]. Furthermore, SREP sensitization showed superior reliability and capacity to discriminate between FMS and RA patients than Temporal Summation of Pain (TSP), a recognized central sensitization index [6].

A pressure algometer Tracker Freedom (JTECH Medical, Lawndale) with a surface stimulation area of 1 cm^2^ was used to measure pain threshold and tolerance and to elicit SREP sensitization [5,6]. To assess pain, a 10-cm visual analogue scale (VAS) was completed after the application of each stimulus, with the anchors being “no pain” and “extremely painful.” Participants received information about the concepts of pain threshold and tolerance and practiced using the VAS. Pain pressure was delivered to the nail of the index finger of the left hand. Pain threshold (the pressure at which the participant started to feel pain) and tolerance (the maximal tolerated pressure) were evaluated at a rate of increase in pressure of 1 kg/s.

The pressure used for the SREP protocol was calculated individually for each participant using the following equation: SREP Intensity = Threshold + 1.25*(DF/4), where DF = Tolerance − Threshold [38]. Participants rested quietly for 10 min before administration of the SREP protocol. For SREP elicitation, a single series of nine painful stimuli, 5 s in duration, was administered according to the individually calibrated pressure. Five seconds after stimulus withdrawal, the VAS was presented. The inter-stimulus interval was approximately 30 s. SREP sensitization was calculated as the difference in pain intensity ratings between the last and first stimulus [5,6]. 

### 2.4. Procedure

The study was conducted over two sessions that took place on the same day. During the first session, clinical histories and socio-demographic data were obtained. It was confirmed that there were no violations of the exclusion criteria. Then, the SCID-I interviews were completed, and self-report instruments were administered. During the second session (laboratory), pain threshold and tolerance were obtained, and the SREP protocol was presented. Participants were asked not to take analgesics drugs for 24 h before the study. No instructions were provided regarding anxiolytics and antidepressants, which could be taken as usual. All participants signed an informed consent form. The study protocol was approved by the Ethics Committee of the University of Jaén (CEIH 160715).

### 2.5. Statistical Analysis

Exploratory factor analysis (EFA) (main component method) was used to explore latent constructs, and confirmatory factor analysis (CFA) (varimax rotation) was employed to confirm the hypothesized factor structure. Eigenvalues > 1 were used to determine significant factors, and a minimum of 2% of explained variance was used as criteria to include a factor in the CFA. The cut-off value for assignment of an item to a factor was set at 0.30 [39]. Items that did not reach this value for any of the factors were removed from the analysis. Internal consistency was analyzed based on Cronbach’s α. Associations of WPI and SS scores with clinical and emotional variables were analyzed through Spearman correlations. Group means were compared by Mann–Whitney U tests. Logistic regression analyses were performed for estimating the diagnostic accuracy of ACR 2010 scales, with group as dependent variable and WPI and SS as predictor variables. WPI and SS were included in the regression model both separately and jointly (using the forward selection method) for differentiating between FM and RA patients. The Fisher’s Z statistic was used to compare accuracy among the models (i.e., WPI, SS and WPI + SS). The sensitivity and specificity values were derived from the classification tables produced in the analyses, with these values defined as follows: Sensitivity = true positives / (true positives + false negatives), and Specificity = true negatives / (true negatives + false positives). Statistical significance was set at *p* < 0.05.

## 3. Results

### 3.1. Demographic and Clinical Data of FMS and RA Patients

Table 1 displays the demographic and clinical data of FMS and RA patients. FMS patients had similar BMI, age, and years of education than RA patients. In addition, FMS patients showed a higher drug intake in all medications, except for non-opioids analgesic, than RA patients and greater emotional comorbidities compared to RA patients. Moreover, FMS patients exhibited higher clinical severity and lower pain threshold and tolerance values. Finally, FMS patients displayed greater SREP sensitization than did RA patients.

### 3.2. Frequency Distribution of WPI and SS Scores

**Figure 1:**
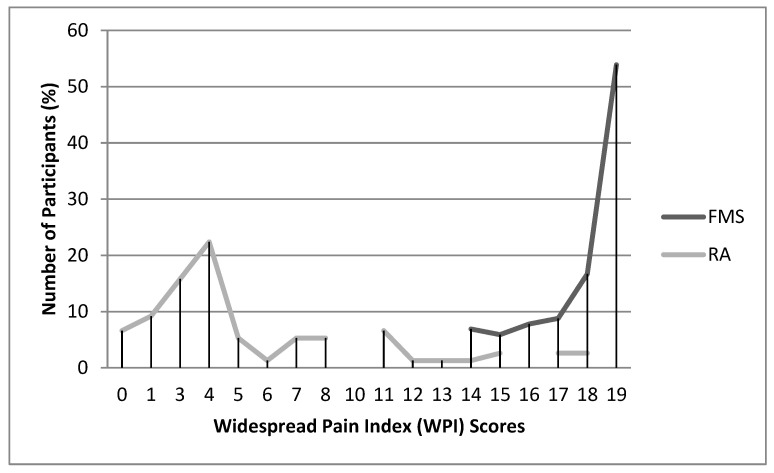
Distribution of Widespread Pain Index (WPI) scores in Fibromyalgia syndrome (FMS) and rheumatoid arthritis (RA) patients.

**Figure 2:**
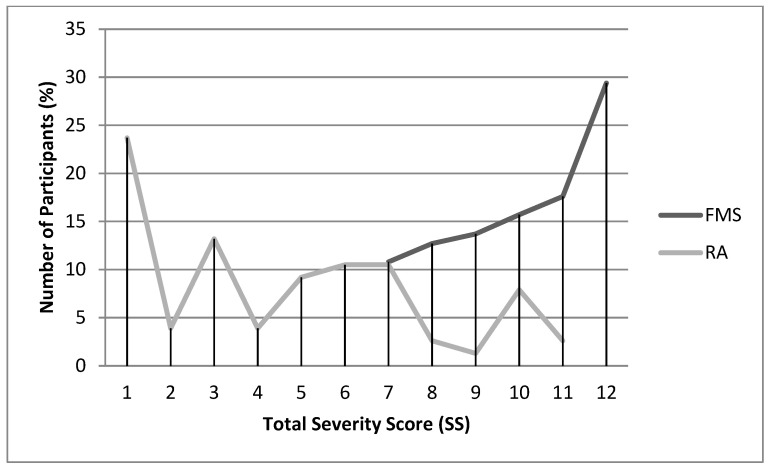
Distribution of Total Symptoms Severity (SS) scores in FMS and RA patients.

Figure 1 and Figure 2 display the frequency distributions of the WPI and SS scores of the FMS and RA patients. Scores were not normally distributed in either the FMS (Kolmogorov–Smirnov test for WPI = 0.30, *p* < 0.001; for SS = 0.18, *p* < 0.001) or the RA patients (Kolmogorov–Smirnov test for WPI = 0.25, *p* < *0*.001; for SS = 0.15, *p* = *0*.001). In FMS patients, scores were negatively skewed (asymmetry for WPI = −1.24, standard error = 0.24; asymmetry for SS = −0.39, standard error = 0.24), with most of the observations being towards the upper end of the scale (19 for WPI and 12 for SS). In RA patients, scores were positively skewed (asymmetry for WPI = 1.20, standard error = 0.29; asymmetry for SS = 0.44, standard error = 0.29), with most of the observations being towards the lower end of the scale. On the SS scale, most FMS patients reported fatigue, cognitive symptoms and waking unrefreshed. In addition, large proportions of FMS patients (53.9%) marked the 19 possible painful areas on the WPI and were assigned to the highest category of 25 or more symptoms on the SS2b (64.7%). In contrast, the majority of the RA patients did not report fatigue, cognitive symptoms, or waking unrefreshed; 60.3% of them indicated between 0 and 4 painful areas on the WPI and 81.6% between 1 and 10 symptoms on the SS2b.

### 3.3. Factor Structure of the WPI and SS

Firstly, we tested the hypothesis of a single-factor structure of the WPI and SS. For this purpose, all items of both scales were subjected to CFA assuming a two-factor solution, i.e., painful body-parts and pain-severity. This hypothesis was not supported, as most of the WPI and SS items did not load onto any of these factors (factor loading cutoff = 0.30). Factor 1 exhibited loads of 2 WPI items and 18 SS items; factor 2 exhibited loads of 1 WPI item and 8 SS items. An EFA of the items of both scales together revealed 22 factors fulfilling the Eigenvalue > 1 criterion, which explained 79.18% of the variance. The WPI and SS items loaded onto different factors without any overlap.

Based on these results, further factor analyses were performed separately for the WPI and SS. The EFA for the WPI item revealed nine factors fulfilling the Eigenvalue > 1 criterion and a minimum of 2% of explained variance, explaining 77.44% of the variance. This 9-factor solution was confirmed by CFA. The nine factors were as follows: (1) shoulder girdle left, shoulder girdle right, and upper arm left; (2) lower arm left and upper and lower back); (3) jaw right and upper leg left; (4) lower arm right and hip buttock left; (5) (chest, abdomen and hip buttock right; (6) upper leg right and lower leg left; (7) jaw left and neck; (8) upper arm right; and (9) lower leg right. Table 2 displays the factor loading matrix according to the CFA (Varimax rotation) of the WPI. Items loading onto more than one factor were assigned to the factor for which the load was higher.

The EFA for the SS revealed 12 factors fulfilling the Eigenvalue > 1 criterion, explaining 70.28% of the variance. Analyzing the scree plot of the sedimentation graph and using the criteria of a minimum of 2% of the variance explained, a 4-factor solution was chosen. The CFA using these four factors explained 41.21% of variance; two items were excluded (vomiting and painful urination). The four factors included were as follows: (1) muscle pain, irritable bowel syndrome, fatigue/tiredness, thinking about or remembering problems, muscle weakness, headache, pain/cramps in abdomen, numbness /tingling, and hair loss; (2) dizziness, insomnia, depression, constipation, pain in upper abdomen, nausea, nervousness, chest pain, blurred vision, fever, diarrhea, and dry mouth; (3) Raynaud’s symptoms, ringing in ears, heartburn, loss/change in taste, shortness of breath, loss of appetite, rash, hearing difficulties, easy bruising, frequent urination, and bladder spasms; and (4) itching, wheezing, hives/welts, oral ulcers, seizures, dry eyes, and sun sensitivity. Table 3 displays the factor loading matrix according to the CFA (Varimax rotation) of the SS.

### 3.4. Internal Consistency of the WPI and SS Scales

The Cronbach’s α (i.e., internal consistency) was 0.34 for the WPI, 0.83 for the SS and 0.82 for both scales combined (WPI + SS). The WPI and SS scores were correlated in the FMS patients (*r* = 0.40, *p* < 0.001) but not in the RA patients (*r* = −0.13, *p* = 0.29).

### 3.5. Associations of the WPI and SS Scores with Clinical Symptoms

Table 4 and Table 5 display the correlations of WPI and SS scores with clinical and emotional variables. The WPI score correlated positively with Total Clinical Pain (MPQ), Current Pain Intensity (MPQ), Emotional Pain (MPQ), Sensory Pain (MPQ), and Insomnia (COS). Total Symptoms on the SS2b and the total SS correlated positively with Total Clinical Pain (MPQ), Current Pain Intensity (MPQ), Emotional Pain (MPQ), Sensory Pain (MPQ), and Fatigue (FSS). In addition, Total Symptoms on the SS2b correlated positively with Depression (BDI), Trait Anxiety (STAI-T), and Catastrophizing (CSQ).

### 3.6. Associations between WPI, SS and Evoked Pain Measures

Only the severity of waking unrefreshed (SS2a) was related to evoked pain, with a negative correlation seen with pain threshold. Total Symptoms (SS2b), Range of Symptoms (SS2b) and Total Symptom Severity (SS) correlated negatively with SREP sensitization. The latter associations were contrary to our predictions. We computed partial correlations to identify possible variables mediating the relationship between SREP sensitization and SS scores. SREP correlated negatively with Fatigue (FSS) (*r* = −0.25, *p* = 0.021) and Depression (BDI) (*r* = −0.31, *p* = 0.003), and SS correlated positively with Fatigue (FSS) (*r* = 0.35, *p* < 0.001) and Depression (BDI) (*r* = *0*.33, *p* = 0.001). After controlling for Fatigue (FSS) and Depression (BDI), SREP sensitization no longer correlated with Total Symptoms (SS2b) (*r* = −0.10, *p* = *0*.34), Range of Symptoms (SS2b) (*r* = −0.19, *p* = 0.08), or Total Symptom Severity (SS) (*r* = −0.09, *p* = 0.42).

### 3.7. Ability of WPI and SS to Differentiate between FMS and RA Patients

All FMS patients, recruited based on the 1990 ACR criteria, fulfilled the 2010 ACR criteria. However, 13 RA patients (19.12% of the sample) fulfilled the 2010 ACR criteria (false positives), resulting in a sensitivity of 100%, specificity of 81%, and overall diagnostic accuracy of 92.4%. The positive predictive value was 88.69%, and the negative predictive value was 100%.

The logistic regression analyses showed that both the WPI (β = −0.73, SE = 0.14, Wald= 26.07, *p* < 0.001) and SS (β = −0.84 SE = 0.13, Wald = 41.17, *p* < 0.001) scores discriminated between the two groups. When the two scales were jointly included as predictors using the forward selection method, the WPI score was selected in the first model. When the scores of both scales were used, discrimination accuracy reached 100%. Table 6 displays the sensitivity and specificity values separately for each predictor and jointly (i.e., for both the WPI and SS).

The Fisher’s Z statistic indicated an increase in diagnostic accuracy between the models for WPI and WPI + SS (Z = 22.73; *p* < 0.001). Furthermore, diagnostic accuracy was greater for WPI than for SS (Z = 4.21; *p* < 0.001).

Using the 2010 ACR criteria, the specificity was 81% but reached 100% when using the complete range of WPI and SS scores in the logistic regression analysis. We analyzed other possible combinations of WPI and SS cut-off values in order to improve specificity. Using WPI ≥14 and SS ≥7, the specificity and overall diagnostic accuracy both reached 100%.

## 4. Discussion

The present study evaluated the reliability, factor structure and predictive validity of the WPI and SS scales of the 2010 ACR criteria for FMS. The mean WPI (17.84) and SS (10.05) scores of our FMS patients were higher than those previously reported. In the 2010 Wolfe et al. study [16], mean scores of 11.4 for WPI and 8 for SS were observed [16]. In a Spanish FMS sample, reported mean scores were 13 for WPI and 8 for SS. Moreover, in Spanish patients, median scores of 15 for WPI and 8 for SS have been reported [26]. A possible explanation for this divergence is that our FMS sample was recruited using the 1990 ACR criteria. This supports the notion that the 1990 criteria are stricter than those of 2010 [16], such that only more severely affected patients are identified.

WPI and SS did not follow a normal distribution. In our FMS patients, the scores were concentrated towards the upper end of the scales (53.9% of them indicated all 19 of the 19 possible painful areas on the WPI, and 64.7% occupied the upper category of 25 or more symptoms on the SS2b); the scores of RA patients were concentrated towards the lower end of the scales (60.3% of them indicated between 0 and 4 painful areas on the WPI, and 81.6% reported between 1 and 10 symptoms in the SS2b). Thus, a strong ceiling effect was observed for the WPI and SS scores in our FMS patients. In accordance with their aim, the scales correctly differentiated between FMS patients and those with AR. The FMS patients indicated more painful areas, increased fatigue, a stronger tendency to wake unrefreshed, more severe cognitive symptoms, and a higher total number of symptoms than those with RA [40].

One of the reasons for developing the 2010 ACR criteria was to devise an instrument differentiating between individual patients or patient groups according to symptom severity [16]. However, the observed ceiling effects impede such differentiation between FMS patients and restrict the utility of the scales for clinical evaluations. Furthermore, the deviation from a normal distribution may complicate the statistical analyses. Therefore, it would be useful to include more elements in both scales (painful areas and symptoms) or to introduce dimensional (Likert scale), instead of categorical, response options to improve the discrimination according to clinical severity of FMS patients.

Contrary to our prediction, neither the WPI nor the SS exhibited a single-factor structure. The WPI items were spread across nine factors covering all painful areas represented in the scale. Most of the factors include two body areas except for Factors 1, 2, and 5, which include three body areas, and Factors 8 and 9, which only include one body area. While it is difficult to explain the loads of different body areas on the same factor, the finding challenges the validity of aggregating pain in different body areas to a single symptom scale. The factor structure of the SS was more intuitive; it involved four factors related to (1) the main clinical FMS symptoms (e.g., muscle pain, fatigue, and cognitive problems), (2) emotional-nervousness symptoms (e.g., depression, anxiety, nausea, nervousness, chest pain, blurred vision, and dry mouth), (3) somatic symptoms (e.g., Raynaud’s symptoms, ringing in ears, heartburn, loss/change in taste, hearing difficulties, and loss of appetite), and (4) symptoms associated with the skin and sensory sensitivity (e.g., itching, wheezing, hives/welts, dry eyes, and sun sensitivity).

The internal consistency of the WPI was poor, which accords with the high number of factors revealed by the EFA and reflects the low co-variation of the item on the instrument (where feeling pain in one body area does not necessarily relate to feeling pain in another body area). In addition, the internal consistency of the scale may be limited by the binary response mode and the small number of items [41]. The SS and the combination of both scales showed satisfactory internal consistency. Most previous studies assessing the internal consistency of the two scales together revealed similar values. While in the present study the Cronbach´s α of the two scales in combination was 0.82, it was 0.87 in the French validation study [20], 0.77 in the Turkish one [21], and 0.93 in a previous Spanish study using the revised 2010 criteria with patient´s self-administration procedure [23]. The WPI and SS scores exhibited a moderate correlation in FMS patients but not in RA patients, suggesting a positive association between the extent of the painful body areas and pain severity.

The WPI and SS demonstrated acceptable predictive validity, as indexed by their associations with clinical symptoms. Despite the skewness of their distributions, making it more difficult to obtain significant correlations, the WPI and the Total Symptoms SS scores correlated positively with the Total Clinical Pain, Current Pain Intensity, Emotional Pain, and Sensorial Pain subscale scores of the MPQ. Similarly, correlations of WPI and SS scores with pain intensity measured by a VAS were demonstrated in previous research [21]. An important symptom of FMS is fatigue [16,42]. Accordingly, SS Fatigue, SS Cognitive Symptoms, the Subtotal SS2a, and SS Total Symptoms scores correlated positively with Fatigue. The SS and WPI scores were also related to emotional variables. In particular, the SS Total Symptoms and Range of Symptoms scores correlated positively with Depression and Catastrophizing. The Total Symptoms and Range of Symptoms scores also correlated positively with Trait Anxiety. This is in line with previous findings of associations between negative emotions and clinical symptoms [43,44]. Several studies have shown that depression and anxiety predispose FMS patients to lifestyles that negatively influence clinical course and psychosocial resources [45,46]. Furthermore, negative emotions are believed play a role in the exacerbation and maintenance of FMS symptoms [43,44]. In a previous study of Spanish patients [23], WPI and SS scores were also significantly associated with depression and fatigue, with the magnitude of the reported associations being greater than that obtained in our study. One potential explanation for the stronger associations found in that previous study is the use of revised 2011 ACR criteria, with the patients´ self-administered method and the somewhat different SS scale (modified for self-application). We believe that self-administration of the scales might enhance the influence of emotional state on symptom and severity reporting [44]. Subjective cognitive symptoms, as assessed by the SS2a correlated positively with Current Pain Intensity, Emotional Pain and Fatigue in this study. This accords with neuropsychological studies indicating that cognitive deficits in FMS vary according to clinical pain severity and fatigue [47,48].

Analysis of associations of WPI and SS scores with indicators of central nervous sensitization to pain is a relevant question, as this is considered the core pathological mechanism of FMS [2,3]. For this purpose, the SREP protocol was used, which was previously demonstrated to be suitable for assessing central pain sensitization in FMS [5,6]. Total Symptoms, Range of Symptoms, and Total Symptom Severity (SS) negatively correlated with SREP. These relations are contrary to our predictions and may be explained by the mediating effects of depression and fatigue, which were associated both with SS and SREP scores. When fatigue and depression were statistically controlled no correlations arose between SS and SREP. The 1990 ACR criteria were criticized due to a lack of associations with measures of evoked pain [49]. In our study, only a significant inverse association with pain threshold was observed for the symptom of waking unrefreshed. This is coherent with previous findings in which FMS patients with poor sleep quality reported greater clinical pain, as well as with the positive association between sleep quality and pain threshold [50]. Nonetheless, it is evident that, just as with the 1990 ACR criteria, WPI and SS scores do not reliably predict responses to evoked pain.

Patients diagnosed with FMS, compared to RA patients, exhibited higher levels of clinical pain, fatigue, anxiety, depression, insomnia and catastrophizing, a lower pain threshold, lower tolerance of pain, and stronger central pain sensitization, which is coherent with current evidence [40]. Regarding the diagnostic accuracy of the 2010 ACR criteria for differentiating between the two patients’ groups, all FMS patients diagnosed with the 1990 ACR criteria also fulfilled the 2010 ACR criteria; there was perfect agreement between both criteria, as previously reported [18]. However, 19.12% of our RA sample fulfilled the 2010 ACR criteria (false positives). In our sample, the 2010 ACR criteria showed 100% sensitivity, as all FMS patients were detected, in addition to good specificity and overall discrimination accuracy. These results contrast with those reported in the previous Spanish study by Segura-Jiménez et al. [23], in which a sensitivity of 88.3% and specificity of 91.8% were obtained in the discrimination between FMS patients and healthy controls. The greater specificity than sensitivity in that study and the greater accuracy in our study in the discrimination between FMS and RA patients, as opposed to the healthy individuals in that study, is difficult to explain. One reason may be the self-administration method used in that study in comparison to the face-to-face interview conducted in the present study. In a Spanish study using the 2010 ACR criteria to differentiate FMS patients from those with RA and osteoarthritis (OA), a sensitivity of 85.6% and specificity of 73.2% were noted [26]. Finally, in the original study of Wolfe at al. [16], an 88.1% of cases were classified correctly. As such, the diagnostic accuracy observed in the present study somewhat exceeded that of previous studies.

The results of our logistic regression analysis showed that WPI and SS also achieved good group discrimination when used separately. However, the WPI was a better predictor than the SS, reflecting the central role of pain in FMS. This is in accordance with previous research [22], which suggested that the WPI is the better single indicator of disease severity and quality of life in FMS. When the two scales were both included in the logistic regression, a diagnostic accuracy of 100% was obtained. The fact that using the 2010 ACR criteria achieved a specificity of 81%, while using the continuous WPI and SS scores led to perfect group discrimination, underlines the potential to improve the diagnostic criteria. Therefore, we tested the effect of using higher cut-off values in order to increase specificity. When cut-off values were set at WPI ≥14 and SS ≥7, both specificity and overall diagnostic accuracy reached 100%. Whereas previous studies proposed different cut-off values [51], cross-cultural differences in the expression and rating of symptoms may explain the different results in terms of sensitivity and specificity among different populations [25,52]. Taken together, the frequency distribution of the WPI and SS scores, the mean WPI and SS scores obtained in our sample, and the better specificity observed when using higher cut-off values as criteria suggest that the cut-off values of both scores should be increased, at least for Spanish populations.

Arnold et al. [53] have recently proposed an alternative strategy for the FMS diagnosis in order to overcome the ACR diagnostic criteria limitations. This alternative is part of the ACTTION-APS Pain Taxonomy (AAPT), developed by The Analgesic, Anesthetic, and Addiction Clinical Trial Translations Innovations Opportunities and Networks (ACTTION) public-private partnership with the U.S. Food and Drug Administration (FDA) and the American Pain Society (APS). The ACTTION-APS Pain Taxonomy is a classification system designed for chronic pain [54], assuming evidence-based diagnostic criteria in a multidimensional framework [55]. In the case of FMS, the AAPT diagnostic proposal comprised five dimensions: (1) Core Diagnostic Criteria (the presence of pain in 6 or more body sites from a total of 9 possible localizations, sleep disturbance, and fatigue), (2) Common Features (e.g., tenderness, cognitive impairments), (3) Common Medical and Psychiatric Comorbidities, (4) Neurobiological, Psychosocial, and Functional Consequences, and (5) Putative Neurobiological and Psychosocial Mechanisms, Risk Factors, and Protective Factors. A study comparing the 2011 and 2016 ACR criteria with the AAPT criteria found considerable agreement between both sets of criteria, although a lower percentage of correct classifications was observed for AAPT criteria than for ACR criteria [56]. Furthermore, a higher prevalence of FMS in the German general population was obtained with AAPT criteria than with ACR 2016 criteria [57]. Thus, AAPT criteria seem to diagnose patients with less symptom severity and fewer pain sites [57]. Further research is required to assess the reliability and validity of the AAPT criteria [58].

As limitation of the study, although we have specially care in excluding patients with RA who showed FMS features, it is difficult to completely discard the possibility of the inclusion in the study sample of some cases with dual RA-FMS pathology [59,60]. Besides, from the comparison of levels of clinical symptoms of our FMS patients with values of previous studies (see above), it is possible that our sample was biased toward more severely affected patients and higher scores on the WPI, SS, and other clinical scales [59,60].

## 5. Conclusions

To sum up, the precision of the WPI and SS for assessing FMS symptoms seems to be limited, as they showed skewed distributions with strong ceiling effects and did not exhibit a single-factor structure. In addition, the internal consistency of the WPI is poor. Nonetheless, the associations of the scales with measures of clinical pain, fatigue, insomnia, depression, and anxiety support their predictive validity with respect to the main symptoms of FMS. In contrast, the scores of both scales were virtually unrelated to sensitivity to evoked pain and central pain sensitization. Finally, both scales exhibited high predictive validity (with respect to discrimination of patient groups), showing very good ability to differentiate between FMS and RA patients. However, at least regarding their application to Spanish samples, it may be necessary to add more painful areas to the WPI and more symptoms to the SS, to reduce ceiling effects and improve discrimination between patients who differ in disease severity. In addition, application of higher cut-off values on both scales may increase the specificity of the diagnoses in Spanish samples.

## Figures and Tables

**Table 1:** Sociodemographic data, clinical variables and questionnaire scores in fibromyalgia syndrome (FMS) (*n* = 102) and RA (*n* = 68) patients (M ± SD or number and %) Results of group comparisons (Mann–Whitney U-tests or χ^2^-test).

	FMS	RA	U / χ^2^	*p*
Age	52.44 ± 7.91	51.73 ± 10.98	3224.5	0.89
Body mass index (BMI)	28.20 ± 5.21	27.73 ± 5.32	3221.0	0.43
Years of Education	10.74 ± 4.27	10.25 ± 4.08	3400.5	0.82
Depression (%)	87 (85.29%)	20 (29.41%)	50.14	*p* < 0.01
Anxiety disorders* (%)	93 (91.18%)	27 (39.71%)	47.11	*p* < 0.01
Antidepressant use (%)	88 (86.27%)	17 (25%)	60.32	*p* < 0.01
Anxiolytic use (%)	85 (83.33%)	20 (29.41%)	45.90	*p* < 0.01
Non-opioids analgesic use (%)	85 (83.33%)	51 (75%)	0.35	0.67
Opiate use (%)	56 (54.90%)	19 (27.94%)	10.48	0.001
Widespread Pain Index pain areas (WPI)	17.84 ± 1.60	5.82 ± 4.86	223.5	*p* < 0.01
Symptom Severity fatigue (SS2a)	2.52 ± 0.54	1.42 ± 1.17	1692.0	*p* < 0.01
Symptom Severity waking unrefreshed (SS2a)	2.43 ± 0.64	1.06 ± 1.24	1502.0	*p* < 0.01
Symptom Severity cognitive symptoms (SS2a)	2.41 ± 0.67	0.94 ± 1.06	1070.0	*p* < 0.01
Subtotal Symptom Severity (SS2a)	7.39 ± 1.47	3.42 ± 2.91	964.5	*p* < 0.01
Total of symptoms (SS2b)	27.27 ± 6.64	4.87 ± 4.08	27.50	*p* < 0.01
Range of Symptoms (SS2b)	2.65 ± 0.48	1.09 ± 0.29	108.0	*p* < 0.01
Total Symptom Severity (SS)	10.05 ± 1.73	4.51 ± 3.05	512.0	*p* < 0.01
Slowly Repeated Evoked Pain (SREP)	1.41 ± 1.31	0.05 ± 0.74	842.5	*p* < 0.01
Threshold (kg)	2.20 ± 1.39	3.40 ± 1.80	1872.5	*p* < 0.01
Tolerance (kg)	6.08 ± 3.16	7.94 ± 2.63	1978.5	*p* < 0.01
Total Pain (MPQ)	79.71 ± 35.23	44.60 ± 33.02	1441.5	*p* < 0.01
Current Pain Intensity (MPQ)	3.66 ± 1.12	2.94 ± 1.07	2231.5	*p* < 0.01
Emotional Pain (MPQ)	11.51 ± 7.21	6.96 ± 7.15	2052.0	*p* < 0.01
Sensorial Pain (MPQ)	51.25 ± 23.77	27.27 ± 22.10	1385.5	*p* < 0.01
State-Anxiety (STAI-E)	23.18 ± 10.42	16.12 ± 13.46	2524.5	*p* < 0.01
Trait-Anxiety (STAI-T)	40.88 ± 13.45	28.99 ± 14.17	865.5	*p* < 0.01
Depression (BDI)	42.69 ± 14.21	16.76 ± 15.29	807.0	*p* < 0.01
Fatigue (FSS)	52.86 ± 9.27	38.01 ± 17.97	1755.0	*p* < 0.01
Insomnia (COS)	36.56 ± 12.12	20.88 ± 14.97	1661.5	*p* < 0.01
Catastrophizing (CSQ)	25.73 ± 11.16	11.97 ± 12.33	1382.0	*p* < 0.01

**Note:** * Anxiety disorders comprise panic disorder, generalized anxiety disorder, phobias and adjustment disorder.

**Table 2:** Factor load matrix for confirmatory factor analysis (CFA) of WPI (Varimax rotation).

WPI	Factors								
	1	2	3	4	5	6	7	8	9
Shoulder girdle, left	0.85								
Shoulder girdle, right	0.87								
Upper arm, left	0.63								
Lower arm, left		0.60							
Upper back		0.81							
Lower back		0.81							
Upper leg left			0.75						
Jaw right			0.69						
Lower arm, right				0.66					
Hip (buttock) left				0.92					
Hip (buttock) right					0.65				
Chest					0.63				
Abdomen					0.76				
Upper leg right						0.84			
Lower leg left						0.55			
Jaw left							0.70		
Neck							0.91		
Upper arm, right								0.81	
Lower leg right									0.90

Note: 72.44% of the variance explained.

**Table 3:** Factor load matrix for CFA of Symptom Severity (SS) scale (Varimax rotation).

SS	Factors			
	1	2	3	4
Dizziness	0.57			
Insomnia	0.65			
Depression	0.69			
Constipation	0.65			
Pain in upper abdomen	0.74			
Nausea	0.68			
Nervousness	0.74			
Chest pain	0.81			
Blurred vision	0.50			
Fever	0.61			
Diarrhea	0.49			
Dry mouth	0.59			
Wheezing	0.38			
Muscle Pain		0.53		
Irritable bowel syndrome		0.38		
Fatigue/Tiredness		0.67		
Thinking or remembering problem		0.74		
Muscle Weakness		0.81		
Headache		0.67		
Pain/cramps in abdomen		0.62		
Numbness/tingling		0.57		
Hair loss		−0.32		
Raynauld’s			0.45	
Ringing in ears			0.51	
Heartburn			0.33	
Loss/change in taste			0.56	
Shortness of breath			0.50	
Loss of appetite			0.65	
Rash			0.46	
Hearing difficulties			0.46	
Easy bruising			0.44	
Frequent urination			0.36	
Bladder spasms			0.33	
Hives/welts				0.31
Itching				0.46
Oral ulcers				0.63
Seizures				0.52
Dry eyes				0.59
Sun sensitivity				0.56

Note: 41.21% of the variance explained.

**Table 4:** Correlations of WPI and SS with SREP, pain threshold, pain tolerance, clinical pain, fatigue and insomnia in FMS patients (*n* = 102).

ACR Diagnostic Criteria	SREP	Threshold	Tolerance	TP	CPI	EP	SP	FSS	COS
WPI	−0.09	−0.01	0.04	0.24 ^+^	0.37 *	0.27 *	0.22 ^+^	0.12	0.23 ^+^
SS Fatigue (SS2a)	−0.16	−0.03	0.05	0.09	0.34 *	0.13	0.06	0.22 ^+^	0.17
SS Waking Unrefreshed (SS2a)	−0.12	−0.21 ^+^	−0.10	0.31 *	0.35 *	0.33 *	0.26 *	0.12	0.11
SS Cognitive Symptoms (SS2a)	−0.08	−0.13	−0.16	0.13	0.19	0.19	0.05	0.22 ^+^	0.19
Subtotal SS (SS2a)	−0.16	−0.14	−0.10	0.26 *	0.40 *	0.32 *	0.19	0.21 ^+^	0.14
Total of symptoms (SS2b)	−0.26 ^+^	−0.12	0.04	0.49 *	0.34 *	0.39 *	0.48 *	0.28 *	0.18
Range of Symptoms (SS2b)	−0.32 *	−0.05	0.06	0.50 *	0.26 *	0.41 *	0.46 *	0.25 ^+^	0.09
Total Symptom Severity (SS)	−0.22 ^+^	−0.11	−0.07	0.34 *	0.42 *	0.35 *	0.27 *	0.24 ^+^	0.15

Note: WPI (Widespread Pain Index), SS (Total Symptom Severity), SREP (Slowly Repeated Evoked Pain), MPQ (McGill Pain Questionnaire): Total Pain (TP), Current Pain Intensity (CI), Emotional Pain (EP) and Sensorial Pain (SP), FSS (Fatigue), COS (Insomnia). ^+^ for *p* < 0.05, * for *p* < 0.01, two-tailed testing.

**Table 5:** Correlations of WPI and SS with anxiety, depression and catastrophizing in FMS (*n* = 102).

ACR Diagnostic Criteria	State-Anxiety (STAI-E)	Trait-Anxiety (STAI-T)	Depression (BDI)	Catastrophizing (CSQ)
Widespread Pain Index pain areas (WPI)	−0.01	0.15	0.19	−0.11
Symptom Severity fatigue (SS2a)	−0.12	0.02	0.03	0.03
Symptom Severity waking unrefreshed (SS2a)	−0.03	0.09	0.23 ^+^	0.22 ^+^
Symptom Severity cognitive symptoms (SS2a)	−0.01	0.11	0.11	0.04
Subtotal Symptom Severity (SS2a)	−0.07	0.11	0.21 ^+^	0.11
Total of symptoms (SS2b)	0.01	0.26 *	0.34 *	0.20 ^+^
Range of Symptoms (SS2b)	−0.10	0.30 *	0.30 *	0.23 ^+^
Total Symptom Severity (SS)	−0.08	0.16	0.22 ^+^	0.14

**Note:**^+^ for *p* < 0.05, ^*^ for *p* < 0.01, two-tailed testing.

**Table 6:** Sensitivity, specificity and overall accuracy (%) for the discrimination between FMS and RA patients for Widespread Pain Index (WPI) and Symptom Severity (SS) scale.

	WPI	SS	WPI + SS
Sensitivity	100	89.2	100
Specificity	89.7	83.8	100
Overall Accuracy	95.9	87.1	100
